# Adrenomedullin in Tumorigenesis and Cancer Progression

**DOI:** 10.3390/ijms26125552

**Published:** 2025-06-10

**Authors:** Hanyi Li, Weijia Yang, Shiqi Wang, Zhihe Zhao, Wangyang Wang, Mingxuan Shi, Yi Li

**Affiliations:** Key Laboratory of Dental Maxillofacial Reconstruction and Biological Intelligence Manufacturing, School of Stomatology, Lanzhou University, Lanzhou 730030, China; lihanyi21@lzu.edu.cn (H.L.); yweijia2023@lzu.edu.cn (W.Y.); wangshq2020@lzu.edu.cn (S.W.); zhaozhh18@lzu.edu.cn (Z.Z.); wwangyang2024@lzu.edu.cn (W.W.)

**Keywords:** adrenomedullin, tumor, vasodilation, signaling pathways

## Abstract

The pathogenesis of cancer is intricately associated with a multitude of factors, and its precise mechanisms continue to be a central focus of rigorous scientific inquiry. Adrenomedullin (ADM), initially characterized as a potent vasodilator, has subsequently been recognized for its diverse biological functions, including roles in angiogenesis, osteogenesis, and immune modulation. Recent studies have shown that ADM, secreted by tumor cells, also plays an important role in regulating immune escape and angiogenesis in the tumor microenvironment, promoting tumor cell proliferation, resisting apoptosis, adapting to anoxic environments, and participating in the process of chemotherapy resistance. Consequently, ADM is implicated in the pathophysiology of various cancers. This review summarizes the essential functions and potential mechanisms of ADM in the occurrence and progression in cancer, and presents the associated therapeutic challenges.

## 1. Introduction

Adrenomedullin (ADM), a peptide hormone, was first identified and isolated from a human pheochromocytoma in 1993 [1]. The designation ‘adrenomedullin’ was assigned due to its initial identification in adrenal medullary tissue, though subsequent studies revealed its widespread expression in other tissues [2,3]. Since its discovery, research on ADM has expanded significantly, revealing that the gene encoding *ADM* is located on human chromosome 11 [3] and is widely distributed across various organs and tissues, including the cardiovascular system, lungs, gastrointestinal tract, and brain [4,5]. The biosynthesis of ADM parallels that of many other hormones, initiating with the synthesis of a larger precursor protein. This precursor is subsequently cleaved into several active peptides, including pro-ADM N-terminal 20 peptide (PAMP), mid-regional pro-ADM (MR-pro-ADM), adrenomedullin-associated peptide, and a glycine-extended 53-amino acid peptide [6]. This 53-amino acid peptide undergoes an enzymatic amidation reaction to form the mature, biologically active ADM, a 52-amino acid peptide [7]. Initially, ADM was characterized as a potent vasodilator [1]. Subsequent investigations have elucidated its multifaceted biological roles, including vascular stability [8], vascular permeability [9], endothelial integrity [10], vasodilation, angiogenesis [11], diuresis [12], as well as antioxidative and anti-inflammatory activities [13], and infection response mechanisms [14]. The biological effects of ADM are mediated through its binding to a receptor complex consisting of the calcitonin receptor-like receptor (CRLR) and specific receptor activity-modifying proteins (RAMPs) [15,16]. Specifically, the ADM1 and ADM2 receptors are formed by the association of CRLR with RAMP2 and RAMP3, respectively, while the combination of CRLR with RAMP1 constitutes the calcitonin gene-related peptide (CGRP) receptor [17].

However, in various pathological states, such as cardiovascular diseases (e.g., hypertension and stroke) and septic shock, ADM expression is markedly elevated, with plasma levels showing a positive correlation with disease severity [10]. This elevation is closely linked to the upregulation of ADM gene expression in tissues, particularly within vascular endothelial cells and smooth muscle cells. Research has demonstrated that oxidative stress, pro-inflammatory cytokines (such as tumor necrosis factor (TNF) and interleukin-1 (IL-1)), angiotensin II, and endothelin-1 can all enhance ADM expression. Additionally, hypoxic conditions have been shown to stimulate ADM synthesis and release [18].

In recent years, an expanding body of research has demonstrated that ADM plays a pivotal role in the initiation and progression of various cancers. ADM is secreted not only by tumor cells but also by relevant stromal cells within the tumor microenvironment (TME), such as macrophages (MCs), mast cells, and endothelial cells. These cells contribute to the secretion of ADM, thereby facilitating the growth and metastasis of malignant tumors [19,20]. As an angiogenic factor, ADM is highly expressed in various tumor tissues, including hepatocellular carcinoma, oral squamous cell carcinoma, and pancreatic cancer. It promotes neovascularization, which ensures an adequate blood supply to the tumor, thus supporting its growth and dissemination [21]. ADM can act as a paracrine or autocrine molecule, promoting tumor cell proliferation, inhibiting programmed cell death, and enhancing tumor cell resistance to apoptosis [22]. Within the tumor immune microenvironment, ADM modulates immune responses by affecting the function of tumor-infiltrating immune cells, thereby facilitating tumor evasion of immune surveillance and promoting tumor growth. Additionally, ADM expression is significantly upregulated in the TME under hypoxic conditions through the hypoxia-inducible factor-1 (HIF-1) pathway, indicating that ADM plays a crucial role in tumor adaptation to hypoxic environments [23,24]. ADM facilitates tumor advancement by influencing angiogenesis, immune evasion, and tumor cell proliferation.

In summary, the role of ADM in tumorigenesis and cancer progression is complex and multifaceted. Consequently, the ADM signaling pathway emerges as a promising target for anti-cancer strategies. This review offers a comprehensive overview of the physiological functions mediated by ADM, including its roles in vasodilation, fluid balance, and immune regulation. Building on this foundation, we further elucidate the pathways and signaling cascades associated with ADM’s involvement in the initiation, progression, and metastasis of various tumor tissues. Our aim is to provide a theoretical framework for targeted ADM therapy in malignant tumors, with the ultimate goal of enhancing the prognosis and survival rates of cancer patients.

## 2. Physiological Functions of ADM

**Figure 1:**
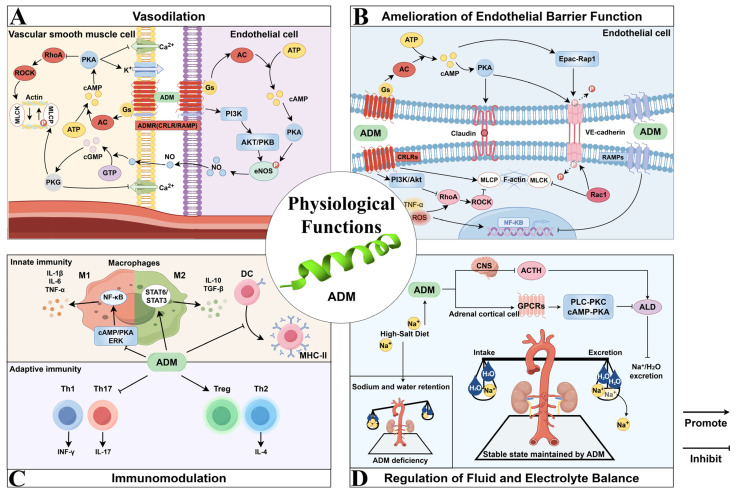
Physiological functions of ADM (this figure is drawn in Figdraw 2.0).

The major physiological functions of ADM include vasodilation, modulation of endothelial barrier function, immunomodulation, and regulation of fluid and electrolyte balance. Figure 1 provides a schematic overview, and the underlying mechanisms for each function are comprehensively described in the ensuing sections.

### 2.1. Vasodilation

The initial physiological effect identified for ADM was vasodilation (Figure 1A), which induces hypotension and decreases peripheral resistance, thereby leading to an increase in cardiac output (CO) [7,25,26]. It has been suggested that ADM’s hypotensive effects are mediated through multiple pathways, encompassing both cyclic adenosine monophosphate (cAMP)-mediated non-endothelium-dependent mechanisms and endothelium-dependent mechanisms involving nitric oxide (NO) and cyclic guanosine monophosphate (cGMP) [27]. Specifically, upon binding to its receptors, ADM activates adenylyl cyclase (AC), which elevates the production of the second messenger cAMP. The increase in cAMP activates protein kinase A (PKA), resulting in the hyperpolarization of the vascular smooth muscle cell membrane and a reduction in intracellular calcium ion concentration through the activation of K-ATP and K-Ca channels, ultimately culminating in vasodilation [8,28,29,30,31]. Moreover, ADM stimulates the activation of endothelial nitric oxide synthase (eNOS), thereby enhancing NO production. This process is potentially mediated through the phosphatidylinositol 3-kinase (PI3K)-protein kinase B (AKT/PKB) signaling pathway. Subsequently, NO diffuses into smooth muscle cells, where it activates guanylyl cyclase, leading to an increase in cGMP levels and promoting vasodilation via endothelium-dependent mechanisms [16,32,33,34]. Additionally, research indicates that ADM modulates cardiovascular function through the central nervous system. In the paraventricular nucleus (PVN) of obese hypertensive (OH) rats, the adipose afferent reflex (AAR) is pivotal, as it enhances sympathetic efferent nerve activity and raises blood pressure. ADM, through its receptor-mediated NO and γ-aminobutyric acid type A (GABA-A) receptor pathway, can mitigate this adipose afferent reflex and sympathetic excitation, thereby effectively lowering blood pressure [35].

### 2.2. Amelioration of Endothelial Barrier Function

ADM also serves as a regulatory agent that stabilizes endothelial barrier function (Figure 1B) [36]. Numerous experimental studies have substantiated the pivotal role of ADM in enhancing endothelial barrier function across various disease models. For instance, in a diabetic macular edema model, ADM significantly decreased retinal vascular permeability and inflammation, thereby demonstrating its protective effects on the retinal endothelial barrier [37]. Similarly, in a sepsis model, ADM notably improved endothelial barrier function and increased survival rates by inhibiting vascular leakage and minimizing organ damage [10,14,38]. In a lipopolysaccharide (LPS)-induced lung injury model, ADM significantly mitigated pulmonary edema and leukocyte extravasation [39]. The protective effects of ADM on the endothelial barrier are primarily mediated through the cAMP signaling pathway, which comprises two principal branches: the PKA pathway and the exchange protein directly activated by cAMP-Rap1 (Epac-Rap1) pathway. ADM specifically strengthens tight junctions and adherens junctions among endothelial cells, thereby minimizing intercellular gaps and decreasing barrier permeability [40]. This is achieved by upregulating the expression of tight junction proteins, such as vascular endothelial cadherin (VE-cadherin), and diminishing their phosphorylation levels.

It was reported that ADM effectively attenuated damage to endothelial cells induced by inflammatory mediators by suppressing the expression of pro-inflammatory cytokines, such as tumor necrosis factor-alpha (TNF-α) and interleukin-1 beta (IL-1β) [15,41,42]. In an LPS-induced acute respiratory distress syndrome (ARDS) mouse model, RAMP2 and RAMP3 were found to play crucial roles inflammatory modulation functions of ADM [43]. These findings further elucidate that ADM can enhance the endothelial barrier function through its anti-inflammatory mechanisms.

ADM is instrumental in safeguarding endothelial cells against apoptosis. In mice lacking RAMP2, an increase in pulmonary cell apoptosis was observed, suggesting that ADM, when coupled with RAMP2, effectively mitigates cell apoptosis [44]. Conversely, RAMP3 is predominantly associated with the regulation of later inflammatory stages. In *RAMP3* knockout mice, no significant alterations in survival rate or lung weight were noted during the initial inflammatory phase following LPS administration. However, there was a marked downregulation of inducible nitric oxide synthase, TNF-α, and the NOD-, LRR-, and pyrin domain-containing protein 3 (NLRP3) inflammasome expression during the later stages of inflammation. This indicates that RAMP3 is crucial in modulating chronic and persistent inflammation.

### 2.3. Immunomodulation

ADM is integral to immunomodulation, serving as an endogenous immunomodulatory factor with pronounced anti-inflammatory properties (Figure 1C). Research has shown that ADM and its receptors are expressed in a variety of immune cells, such as macrophages, monocytes, and T cells, and are also present in lymphoid organs and the gastrointestinal tract [45]. ADM modulates the immune system by influencing the activity of immune cells and the cytokines they produce, thus attenuating the excessive activation of the inflammatory response [10,42].

ADM exerts substantial immunomodulatory effects on macrophages, notably influencing their polarization states. Macrophages have the capacity to differentiate into M1 (pro-inflammatory) and M2 (anti-inflammatory) phenotypes. Empirical studies have demonstrated that ADM can modulate macrophage polarization in response to bacterial LPS, facilitating a shift from the M1 to the M2 phenotype. This regulation is achieved through metabolic reprogramming, wherein ADM promotes a transition in macrophage metabolism from glycolysis to mitochondrial oxidative phosphorylation [46,47].

Moreover, ADM demonstrates its role in immunomodulation by suppressing the synthesis of pro-inflammatory cytokines. In a rat model of interstitial cell inflammation induced by IL-1β, ADM was shown to decrease the expression of the pro-inflammatory factor receptor activator of nuclear factor-kappa B ligand (RANKL), which is induced by IL-1 and TNF-α, through the inhibition of extracellular signal-regulated kinase (ERK) and p38 mitogen-activated protein kinase pathways [48]. The equilibrium between pro-inflammatory and anti-inflammatory factors is essential for the proper regulation and resolution of inflammatory responses [49,50]. Under normal physiological conditions, ADM modulates immunomodulatory responses by regulating the expression and release of inflammatory cytokines [42]. This regulation contributes to maintaining the body’s immune homeostasis.

### 2.4. Regulation of Fluid and Electrolyte Balance

ADM is integral to the regulation of fluid and electrolyte homeostasis [51]. Research utilizing rat models has demonstrated that prolonged high-salt consumption results in elevated ADM concentrations within the adrenal glands and kidneys, suggesting ADM’s critical involvement in maintaining the body’s salt and water equilibrium [52,53]. ADM plays a critical role in the regulation of fluid and electrolyte balance through various mechanisms. Within the central nervous system (CNS), ADM inhibits the secretion of adrenocorticotropic hormone (ACTH), leading to a reduction in aldosterone secretion [7,54]. This reduction subsequently diminishes sodium retention and water reabsorption, thereby influencing fluid homeostasis. Additionally, ADM exerts a direct effect on adrenal cortical cells by binding to specific receptors on the cell surface, predominantly G protein-coupled receptors (GPCRs). This process involves the cAMP-PKA and phospholipase C-protein kinase C (PLC-PKC) signaling pathways, which not only reduces the synthesis and secretion of aldosterone, but also plays a further role by regulating the expression of genes related to aldosterone production, which may involve the activation or inhibition of transcription factors. Furthermore, adrenal cortical cells exhibit high sensitivity to alterations in blood flow, with increased perfusion potentially further inhibiting their activation and subsequent aldosterone secretion.

In conclusion, ADM is integral to the regulation of fluid and electrolyte balance. Its effects extend across the central nervous system, kidneys, and endocrine system, operating through the synergistic interplay of various mechanisms (Figure 1D).

## 3. Impact of ADM on Tumors

**Figure 2:**
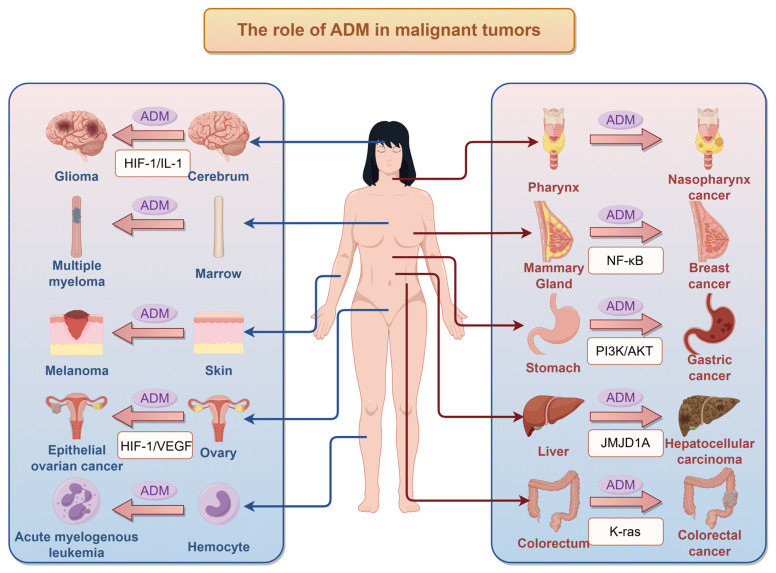
The role of ADM in tumors (this figure was drawn in Figdraw 2.0).

The expression of ADM demonstrates intricate characteristics within tumor tissues. Notably, ADM is aberrantly overexpressed in various tumor cell lines and solid tumors, where it plays a significant role in promoting tumor angiogenesis and stimulating cellular proliferation. The role of ADM in related tumors is summarized in Figure 2. Research indicates that both mRNA and protein levels of ADM are markedly elevated in breast cancer tissues, with a strong correlation to low histological differentiation and the presence of axillary lymph node metastasis [55,56]. As breast cancer progresses, characterized by increasing tumor size and advancing clinical stage, serum levels of ADM in patients also rise correspondingly [19]. In lung cancer tissues, the positive expression rate of ADM is observed to be 70%, which is significantly higher than that in normal lung tissue, and its expression is associated with pathological type, clinical stage, and lymph node metastasis [57,58]. Conversely, in triple-negative breast cancer, ADM expression is significantly lower compared to that in healthy tissue. Patients exhibiting low levels of ADM are at an increased risk of recurrence and metastasis, often presenting with more severe disease and a poorer prognosis [55]. Additionally, ADM expression is found to be diminished in pituitary adenomas when compared to non-neoplastic pituitary glands [59].

**Figure 3:**
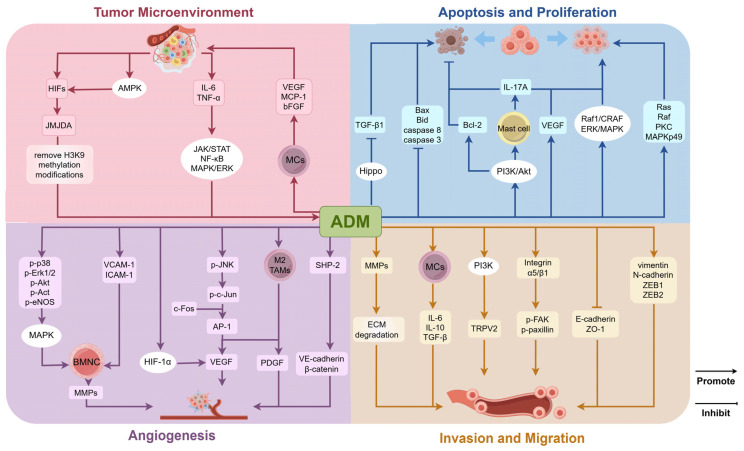
The mechanism of action of ADM on tumor cells (this figure is drawn in Figdraw 2.0).

The variability in ADM expression observed in tumors can be attributed to multiple factors. Primarily, characteristics of the tumor microenvironment, including hypoxia and nutrient deprivation, modulate ADM expression by activating pathways such as HIFs. Additionally, inflammatory cytokines like IL-6 and TNF-α may affect ADM synthesis through specific signaling pathways [27]. Furthermore, the regulation of ADM expression is governed by a complex network of gene modifications and interactions among signaling molecules. The influence of these mechanisms may vary across different tumor types. Understanding the regulation and mechanisms of ADM action in malignant tumors offers a novel perspective on tumor biology and provides a theoretical foundation for potential clinical applications or the development of therapeutic strategies. The involved mechanisms of ADM on tumor cells are summarized in Figure 3. Several aspects of impacts of ADM on tumors are mainly considered, including roles of ADM in tumor microenvironment, tumor cell proliferation and apoptosis, angiogenesis in tumor tissues, and tumor cell invasion and migration capabilities.

### 3.1. Role of ADM in the Tumor Microenvironment

The TME represents a highly intricate and dynamically evolving ecosystem that is pivotal in the initiation and progression of tumors. It comprises a diverse array of cellular and extracellular constituents, including tumor cells, cancer-associated fibroblasts, immune cells, vascular cells, and the extracellular matrix. The TME is often characterized by hypoxic and acidic conditions, primarily due to insufficient blood supply associated with rapid tumor growth and the accumulation of metabolic byproducts [60]. This milieu not only supports the survival of cancer cells but also fosters the development of drug resistance, such as diminished responsiveness to chemotherapeutic agents [61]. The unique attributes of the TME contribute to the upregulation of ADM expression via multiple signaling pathways, while ADM, in turn, performs significant regulatory functions within this environment through various mechanisms.

The TME is frequently characterized by hypoxia, a condition that arises due to the uncontrolled and rapid proliferation of malignant tumors, which surpasses the capacity of the vascular supply [60]. This imbalance inhibits oxygen diffusion beyond a distance of 100–150 μm, thereby establishing hypoxic conditions. Hypoxia serves as a selective pressure in solid tumors, favoring apoptosis-resistant cell populations and driving tumor evolution toward a more malignant phenotype. Under these conditions, HIFs are activated and subsequently regulate the expression of a series of genes associated with tumor survival and adaptation, including ADM. HIFs modulate ADM expression through multiple pathways, facilitating the adaptation of tumor cells to hypoxic environments. For instance, HIF activation can enhance the expression of the histone demethylase JMJD1A. Once upregulated under hypoxic conditions, JMJD1A can remove H3K9 methylation modifications on the ADM promoter, thereby increasing the transcriptional activity of the ADM gene [62]. Moreover, glucose deprivation, a common phenomenon in metabolically demanding tumors, leads to the activation of AMP-activated protein kinase (AMPK), which subsequently augments HIF-1 activity [63]. This cascade promotes the upregulation of ADM expression. Additionally, inflammatory cytokines, including IL-6 and TNF-α, present in the TME, significantly enhance ADM gene expression. This enhancement is mediated through the activation of transcription factors and downstream signaling pathways, such as Janus kinase/signal transducer and activator of transcription (JAK/STAT), nuclear factor kappa-light-chain-enhancer of activated B cells (NF-κB), and mitogen-activated protein kinase/extracellular signal-regulated kinase (MAPK/ERK).

Previous studies have demonstrated that ADM facilitates communication between tumor cells and MCs. At sub-nanomolar concentrations, ADM induces the migration of MCs toward tumor cells, leading to MC infiltration within and around the tumor mass. In these differentiated MCs, ADM expression is further upregulated in response to hypoxic conditions, thereby establishing autocrine and paracrine interactions with the surrounding cellular microenvironment, including tumor cells [64]. As ADM concentrations reach nanomolar levels or higher, there is an upregulation in the expression of angiogenic factors such as the vascular endothelial growth factor (VEGF), monocyte chemoattractant protein-1 (MCP-1), and basic fibroblast growth factor (bFGF) within the MC/tumor complex. These factors are subsequently released into the surrounding microenvironment, promoting tumor progression and angiogenesis. Furthermore, ADM produced by MCs enhances the proliferation and clonogenic potential of tumor cells.

In recent years, scholarly attention has increasingly been directed toward the previously underexplored population of adipocytes within the tumor microenvironment. Accumulating evidence indicates that direct interactions between adipocytes and tumor cells are pivotal in facilitating malignant tumor progression. For example, in the context of breast cancer research, ADM has been shown to stimulate adipocytes, leading to the upregulation of uncoupling protein 1 (UCP1) expression and the enhanced phosphorylation of hormone-sensitive lipase (HSL), which in turn activates lipolysis. Thus, ADM appears to play a critical role in modulating the interactions between cancer cells and adipocytes, suggesting that it may serve as a promising therapeutic target for disrupting these interactions [65].

### 3.2. Regulation of Tumor Cell Proliferation and Apoptosis

ADM exerts a significant impact on the biological behavior of tumor cells, including malignant proliferation, through the modulation of intercellular signaling pathways. In the majority of tumors, ADM expression is elevated compared to that in healthy tissues, exhibiting properties that promote tumor cell proliferation and inhibit apoptosis. For example, studies have shown that RNA-targeted interference with ADM can trigger apoptosis and suppress the growth of bladder urothelial cancer cells [66]. In studies of ovarian cancer, the suppression of ADM gene expression has been shown to significantly inhibit cell proliferation and enhance chemosensitivity [67]. Across various tumor tissues, ADM regulates tumor cell proliferation and apoptosis via diverse mechanisms, and in breast cancer, cells with ADM overexpression demonstrated a more pleomorphic morphology, an increased potential for angiogenesis both in vitro and in vivo, and a reduction in apoptosis following serum deprivation [19]. For instance, the human breast cancer cell line T47D cells overexpressing ADM exhibited elevated levels of proteins associated with oncogenic signaling pathways, including Ras, Raf, PKC, and MAPKp49, alongside decreased levels of pro-apoptotic proteins such as B-cell lymphoma-2 (Bcl-2)-associated X protein (Bax), Bid, and caspase-8 [68]. In hepatocellular carcinoma (HCC) and endometrial cancer, ADM contributes to resistance against hypoxic apoptosis and fosters cell proliferation by upregulating the anti-apoptotic protein Bcl-2 [69,70]. In gastric cancer (GC) cells, ADM silencing decreases the expression of phosphor-AKT (p-AKT), inhibiting the AKT signaling pathway, downregulating the anti-apoptotic protein Bcl-2, and upregulating the pro-apoptotic proteins cleaved-caspase-3 and Bax, which significantly reduces GC growth and promotes GC cell apoptosis [71]. In addition, another study on GC proposed an alternative mechanism: tumor-derived ADM activates the PI3K/AKT signaling pathway in the GC microenvironment, inducing the degranulation of tumor-associated mast cells to release IL-17A in a dose-dependent manner, thereby promoting GC cell proliferation and inhibiting GC cell apoptosis [72]. In vitro studies of osteosarcoma have demonstrated that downregulation of ADM expression can inhibit cell proliferation via the VEGF pathway [73]. In renal cell carcinoma (RCC) cells, treatment with ADM resulted in the upregulation of ERK1/2 expression, thereby enhancing cancer cell proliferation through the ERK/MAPK signaling pathway [74]. A study on malignant pleural mesothelioma demonstrated that ADM induces cell proliferation in vitro through the activation of the Raf1 proto-oncogene serine/threonine kinase (c-Raf)/mitogen-activated protein kinase 1 (MEK)/ERK pathway [75]. Additionally, in investigations concerning Leydig cell steroidogenic function, ADM demonstrates an anti-apoptotic effect by inhibiting transforming growth factor-β1 (TGF-β1) within the Hippo signaling pathway. The Hippo signaling pathway is a frequently dysregulated pathway in cancer, playing a pivotal role in regulating cell proliferation, differentiation, apoptosis, and tissue microenvironment homeostasis [76,77].

### 3.3. Regulation of Angiogenesis in Tumor Tissues

Angiogenesis represents a fundamental characteristic of tumor development and metastasis. While angiogenesis supports tumor proliferation, tumor-associated vasculature is often characterized by significant disorganization, structural abnormalities, and functional deficiencies compared to normal blood vessels, primarily due to the overproduction of pro-angiogenic factors [78]. ADM not only facilitates tumor angiogenesis but also promotes the normalization of tumor vascular architecture, thereby improving the functional blood supply to tumor tissues.

ADM can promote the secretion and expression of pro-angiogenic factors in tumor tissues through multiple mechanisms. The research on pancreatic cancer revealed that treatment with ADM resulted in the phosphorylation of p38, Erk1/2, AKT, and eNOS, thereby enhancing the migration and invasion of bone marrow monocytes through the activation of the MAPK, PI3K/AKT, and eNOS signaling pathways, as well as increasing the levels of matrix metalloproteinases-2 (MMP-2) [79]. Additionally, ADM facilitated the adhesion and transendothelial migration of bone marrow monocytes by upregulating the expression of vascular cell adhesion molecule-1 (VCAM-1) and intercellular adhesion molecule-1 (ICAM-1) in endothelial cells. Therefore, ADM promotes tumor angiogenesis by directly recruiting bone marrow monocytes into tumor tissues and modulating the phenotype of myeloid cells, which in turn enhances the secretion of angiogenic factors [79]. Furthermore, in the study of pancreatic ductal adenocarcinoma, ADM influences macrophages and myeloid-derived suppressor cells (MDSCs) to adopt phenotypes conducive to tumor growth, characterized by the secretion of various pro-angiogenic factors. In the investigation of epithelial ovarian cancer (EOC), there is evidence demonstrating that ADM functions as an upstream regulatory molecule of VEGF and HIF-1α, thereby facilitating tumor angiogenesis through the upregulation of VEGF and HIF-1α expression [67,80]. Additional research has demonstrated that ADM facilitates the phosphorylation of its downstream target protein, c-Jun, by enhancing the phosphorylation of c-Jun N-terminal kinase (JNK). The protein c-Jun associates with c-Fos to form the activator protein 1 (AP-1) transcription factor, which can bind to the promoter region of VEGF. Consequently, ADM is capable of inducing VEGF expression in cancer cells via the JNK/AP-1 signaling pathway, thereby promoting cancer progression through the enhancement of tumor angiogenesis [81].

Solid tumors are not exclusively composed of cancerous cells; they also encompass non-malignant resident stromal cells, including bone marrow-derived cells (BMDCs) and cancer-associated fibroblasts (CAFs) [82,83]. Among the BMDCs, tumor-associated macrophages (TAMs) constitute the most prevalent component, characterized as macrophages that inhabit the tumor microenvironment. Typically, tumor cells facilitate the polarization of TAMs towards an M2-like phenotype, which is conducive to enhancing tumor growth, angiogenesis, invasion, and metastasis [84]. ADM increases the proportion of M2 TAMs through an autocrine mechanism, and M2 TAMs are capable of secreting a variety of pro-angiogenic factors, such as VEGF, MMPs, and platelet-derived growth factor (PDGF), which promote tumor angiogenesis through both direct and indirect mechanisms [85,86]. Additionally, M2 TAMs release anti-inflammatory cytokines, including IL-10 and TGF-β, which suppress the activity of anti-angiogenic immune cells, such as T-helper 1 (Th1) cells and cytotoxic T cells, thereby establishing an immunosuppressive environment conducive to angiogenesis [84]. TAMs play a crucial role in regulating tumor angiogenesis by transferring molecules, such as miRNAs, via exosomes. For instance, miR-301a-3p, transported by exosomes from esophageal squamous cell carcinoma (ESCC) cells, enhances the pro-angiogenic activity of TAMs, thereby facilitating tumor angiogenesis [87]. Another significant stromal cell type, CAFs, exhibit distinct phenotypic and functional characteristics compared to normal fibroblasts, attributed to their increased proliferation rates and differential expression of extracellular matrix (ECM) components and growth factors [88]. In breast cancer research, it has been demonstrated that ADM is among the factors derived from CAFs that facilitate the recruitment of endothelial-like cells and pericytes for neovascularization. Furthermore, ADM contributes to the proliferation of CAFs by extending the phosphorylation of ERK1/2 and activating the MAPK signaling pathway. Additionally, mast cells also serve as crucial mediators of angiogenesis [19]. Within the context of RCC research, it has been demonstrated that cancer cells can modulate glycogen synthase kinase-3β (GSK3β) activity via the PI3K/AKT signaling pathway. This modulation leads to an upregulation of ADM expression, which subsequently recruits mast cells and enhances the migration of vascular endothelial cells towards RCC, thereby promoting angiogenesis in RCC [88].

ADM is pivotal in the normalization of tumor vascular architecture. A fundamental difference between tumor angiogenesis and normal angiogenic processes is that the vessels formed in tumors are tortuous, irregularly shaped, and exhibit high permeability, characteristics indicative of pathological angiogenesis. In the tumor microenvironment, endothelial cells lose tight junctions and adhesion molecules, become highly motile, and infiltrate the interstitial tissue. The ADM-RAMP2 system is essential for vascular development. Research has shown that mice with a homozygous knockout of either *ADM* or *RAMP2* (*ADM*−/− or *RAMP2*−/−) experience embryonic lethality due to vascular abnormalities. The ADM-RAMP2 system plays a crucial role in maintaining vascular integrity, and a deficiency in RAMP2 increases vascular permeability in primary lesions by impairing vascular structural integrity and inducing inflammation. In murine models, this deficiency has been associated with significant vascular narrowing, irregular vascular wall morphology, localized thinning, and partial fragmentation, ultimately leading to vascular leakage [89,90]. Research on glioblastoma has demonstrated that ADM facilitates the activation of Src homology phosphotyrosyl phosphatase 2 (SHP-2) through the phosphorylation of critical residues. This process results in the dephosphorylation of p-VE-cadherin, which subsequently enhances the interaction between VE-cadherin and the β-catenin complex. Ultimately, this mechanism regulates intercellular junctions during angiogenesis, thereby promoting the development of a stable and functional tumor vascular system [91]. Another experiment has demonstrated that at the molecular level, the blockade of ADM induces the phosphorylation of VE-cadherin through the activation of Src kinase, thereby enhancing the interaction between Src and VE-cadherin, which subsequently increases endothelial cell permeability and contributes to the collapse and regression of neovascularization in tumors [92].

### 3.4. Regulation of Invasion and Migration Capabilities

Research has demonstrated that ADM can augment the invasiveness and migratory potential of tumor cells across various neoplastic tissues, including bladder cancer, pancreatic cancer, astroglioma, and ovarian cancer [93,94]. ADM facilitates tumor cell invasion and migration through diverse mechanisms, thereby creating conducive conditions for tumor progression.

MMPs constitute a class of proteases that are capable of degrading components of the extracellular matrix. The extracellular matrix, which is composed of various proteins and polysaccharides, functions as an extracellular scaffold that restricts cellular movement [95]. ADM facilitates extracellular matrix degradation by upregulating the expression and activity of MMPs, thereby enabling tumor cells to penetrate the matrix barrier and disseminate into adjacent tissues [79]. In colorectal cancer studies, ADM expression has been positively correlated with MMP-9 expression, and their combined activity has been shown to enhance the migration and invasion of colorectal cancer cells [96].

In research pertaining to epithelial ovarian cancer, it has been established that tumor-derived ADM can induce macrophage phenotypes analogous to TAMs and stimulate cytokine production. Experimental findings further demonstrated that macrophages facilitate cytoskeletal rearrangement by activating the Ras homolog family member A (RhoA) signaling pathway, thereby enhancing cancer cell migration. In a separate epithelial ovarian cancer investigation, ADM was found to promote cancer cell migration through the activation of the integrin α5/β1 signaling pathway. ADM upregulates integrin α5 expression, subsequently inducing the phosphorylation of focal adhesion kinase (FAK) and paxillin, which in turn promotes cancer cell migration. In studies focusing on prostate cancer and urothelial carcinoma, ADM treatment was shown to enhance cell adhesion and migration via β1-integrin and FAK activation. Additionally, ADM was demonstrated to influence the translocation of transient receptor potential vanilloid 2 (TRPV2) to the plasma membrane through PI3K pathway, leading to increased resting calcium levels in tumor cells, thereby contributing to its effect on tumor cell migration [97].

Epithelial–mesenchymal transition (EMT) is widely recognized as a pivotal mechanism in cancer metastasis. Investigations into intrahepatic cholangiocarcinoma have demonstrated that stable *ADM* transfectants exhibit a marked upregulation of mesenchymal markers, such as vimentin and neural cadherin (N-cadherin), as well as epithelial-mesenchymal transition-associated transcription factors ZEB1 and ZEB2. Conversely, there is a significant downregulation of epithelial markers, including epithelial cadherin (E-cadherin) and ZO-1. In contrast, *ADM*-silenced cells display an inverse expression pattern of these genes [98].

## 4. Drug Resistance and ADM-Targeted Therapies

ADM has been extensively documented in numerous studies as being intricately linked to tumor drug resistance, playing a significant role in the modulation of drug resistance across various cancer types. The following research findings substantiate this association: (1) In renal tumors exhibiting resistance to the anticancer drug sunitinib, there is an upregulation of ADM expression. Inhibition of ADM has been shown to suppress tumor growth, underscoring ADM’s critical involvement in sunitinib resistance [74]. (2) ADM facilitates the proliferation of ovarian cancer cells and impedes apoptosis through the reprogramming of glucose metabolism, thereby augmenting resistance to cisplatin. (3) In gliomas resistant to temozolomide (TMZ), ADM expression is elevated, and the attenuation of ADM levels has been observed to enhance TMZ efficacy, promote apoptosis, and decrease mitochondrial membrane potential [99]. These findings highlight the complex role of ADM in tumor drug resistance. Current research still focuses on fundamental experiments, therefore further investigation and validation are necessary to translate these insights into clinical applications.

ADM is intricately associated with the malignant characteristics and drug resistance observed in tumors. A growing body of research indicates that targeting ADM and its associated signaling pathways represents a promising strategy for inhibiting tumor development by preventing angiogenesis, proliferation, and migration. The blockade of ADM signaling pathways or its receptors using specific antibodies has been shown to effectively suppress tumor growth and metastasis. Currently, a range of strategies have been employed to target the ADM system in cancer treatment. These strategies include: (1) neutralization of the ADM ligand itself; (2) antagonism of the ADM receptor complex, specifically the calcitonin receptor-like receptor (CLR) in combination with RAMP2 or RAMP3; (3) antagonism of individual components of the ADM receptor, namely CLR, RAMP2, or RAMP3. Additionally, direct downregulation of *ADM* gene expression through gene editing techniques, such as RNA interference or the CRISPR-Cas9 system (clustered regularly interspaced short palindromic repeats-associated protein 9), may exert anti-tumor effects by modulating the proliferation and invasiveness of tumor cells. For instance, in glioblastoma treatment studies, the neutralization of ADM with specific antibodies led to a marked reduction in tumor growth and a decrease in vascular density within tumors in animal models [100]. As previously noted, resistance to TMZ represents a significant therapeutic challenge in glioma treatment, whereas strategies aimed at targeting ADM demonstrate considerable efficacy. Recent studies suggest that miR-1297 functions as an upstream regulatory micro-RNA of ADM, directly targeting and inhibiting its expression, and the overexpression of miR-1297 has been shown to enhance the therapeutic effects of TMZ on glioma cells [99]. Similarly, experimental research on melanoma has demonstrated both in vitro and in vivo that antibodies neutralizing ADM and its receptors substantially inhibit tumor cell proliferation, migration, and invasion, culminating in tumor regression [11]. ADM_22-52_, an ADM antagonist, functions by competing with ADM for receptor binding and has been utilized in various tumor models, such as breast cancer, pancreatic cancer, and mesothelioma, to counteract ADM activity. It demonstrates inhibitory effects on cell proliferation, angiogenesis, migration, and invasion [101]. For instance, in an RCC mouse xenograft model, ADM_22-52_ effectively inhibited the growth of sunitinib-resistant tumors, and the combined treatment of RCC cells with sunitinib and ADM_22-52_ proved more efficacious than monotherapy with either agent [74]. Recent studies have focused on the development of chimeric and bispecific antagonists targeting CLR/RAMP receptors, including chimeric analogues composed of acylated truncated ADM analogues, somatostatin analogues, and ADM antagonists. Compared to antibody-based therapies, these analogues exhibit a higher propensity to penetrate the tumor microenvironment, potentially leading to the advancement of improved anti-CGRP therapies and alternative anti-angiogenic treatments [102].

Given that the ADM1 receptor (ADM1R) predominantly regulates blood pressure, clinical inhibition of the ADM1R pathway is deemed unacceptable. In contrast, ADM2R is recognized as a viable target for cancer therapy. Research has elucidated the design, synthesis, and characterization of potent small molecule antagonists of ADM2R, exhibiting a selectivity that is 1000-fold greater than that of ADM1R. These compounds demonstrate significant effects on markers associated with pancreatic cancer progression in vitro, possess favorable pharmacokinetic profiles typical of drug-like substances, and effectively inhibit the growth of xenograft tumors while extending the lifespan of mouse models of pancreatic cancer [103].

In the preceding discussion, it was elucidated that the role of ADM in facilitating tumor progression is contingent upon multiple signaling pathways. Consequently, beyond the therapeutic strategy of directly targeting ADM and its receptor system, the inhibition of associated signaling pathways can also yield anti-tumor effects. For instance, the hypoxic conditions characteristic of the tumor microenvironment result in the upregulation of ADM expression via HIF-1, suggesting that targeting HIF-1 could impede tumor progression. Research pertaining to breast cancer has demonstrated that HIF-1α is upregulated following ATP treatment and plays a role in mediating ATP-induced chemotherapy resistance. The STAT3-ALDOA (aldolase A) axis mediates ATP-HIF-1α signal transduction and enhances the expression of the HIF-1 target gene *ADM*. Treatment with HIF inhibitors in conjunction with the ATP hydrolase apyrase has been shown to increase drug sensitivity in murine models [104]. Furthermore, as previously noted, in the RCC mouse model, ADM upregulates cAMP and activates the ERK/MAPK pathway, thereby promoting cellular proliferation. Experimental findings indicate that, in comparison to the control group, the administration of PD98059, a MAPK kinase inhibitor, effectively inhibits tumor growth [74].

In summary, considering the high expression of ADM in the majority of tumors and its significant association with tumor progression and poor prognosis, therapeutic interventions targeting ADM and its associated signaling pathways have demonstrated substantial anti-tumor efficacy. This approach represents a promising therapeutic strategy. However, current trials are still in the preclinical stage and further research is needed to fill clinical research gaps.

## 5. Conclusions

ADM plays a crucial role in regulating blood pressure, angiogenesis, and immune modulation in normal tissues, and existing studies suggest that ADM is a key molecule in tumorigenesis and progression. This review summarizes the contribution of ADM to tumorigenesis and development and its potential functions in cancer therapy. ADM facilitates tumor cell adaptation to hypoxic conditions within TME, stimulates proliferation, and drives malignant progression by promoting phenotypic transition toward aggressive variants. Concurrently, it induces the formation of structurally abnormal tumor-associated vasculature and enhances invasive capacity through matrix remodeling. While the involvement of ADM in cancer pathogenesis has been extensively investigated, there remain many gaps in the literature on the subject. For instance, ADM exhibits context-dependent modulation of oncogenic signaling pathways across different tumor types, and its pro-tumorigenic mechanisms remain incompletely characterized. These limitations directly impact therapeutic development: current strategies targeting ADM-associated signaling pathways often demonstrate insufficient cancer cell specificity, resulting in off-target effects and potential toxicity to normal tissues. Furthermore, clinical observations reveal a paradoxical pattern in certain malignancies—reduced ADM expression correlates with increased risks of recurrence, metastasis, and poor prognosis. This dualistic behavior fundamentally challenges the therapeutic rationale for universal ADM inhibition and necessitates tumor subtype-specific approaches.

Therefore, we propose that in future research, the use of single-cell transcriptomics and CRISPR interference screening mechanisms is crucial in elucidating the context-dependent role of ADM in different cancer subtypes, particularly its interactions with receptor isoforms and immune suppression pathways. A better understanding of the relationship between ADM and cancer can provide a foundation for clinical applications and open up new strategies for treating cancer patients by targeting ADM. Future research should also prioritize the development of spatially controllable targeted delivery systems to reduce off-target effects or the development of bispecific antibodies targeting both ADM and tumor-specific antigens to precisely design different tumor treatment drugs and enhance tumor-selective efficacy. Further, combining anti-ADM therapy with chemotherapy or immunotherapy should focus on disrupting ADM-mediated immune suppression to guide adaptive treatment strategies.

## Abbreviations

The following abbreviations are used in this manuscript:

AAR	adipose afferent reflex
AC	adenylyl cyclase
ACTH	adrenocorticotropic hormone
ADM	adrenomedullin
ADMR	adrenomedullin receptor
AKT (PKB)	protein kinase B
ALDOA	aldolase A
AMPK	AMP-activated protein kinase
AP-1	activator protein 1
ARDS	acute respiratory distress syndrome
bFGF	basic fibroblast growth factor
CAFs	cancer-associated fibroblasts
cAMP	cyclic adenosine monophosphate
cGMP	cyclic guanosine monophosphate
CGRP	calcitonin gene-related peptide
CLR	calcitonin receptor-like receptor
CNS	central nervous system
CO	cardiac output
CRLR	calcitonin receptor-like receptor
DC	dendritic cell
E-cadherin	epithelial cadherin
ECM	extracellular matrix
EMT	epithelial–mesenchymal transition
eNOS	endothelial nitric oxide synthase
EOC	epithelial ovarian cancer
Epac-Rap1	exchange protein directly activated by Rap1
ERK	extracellular signal-regulated kinase
ESCC	esophageal squamous cell carcinoma
FAK	focal adhesion kinase
GABA-A receptor	γ-aminobutyric acid type A receptor
GC	gastric cancer
GPCRs	G protein-coupled receptors
GSK3β	glycogen synthase kinase 3β
HCC	hepatocellular carcinoma
HIF	hypoxia-inducible factor
HSL	hormone-sensitive lipase
ICAM-1	intercellular adhesion molecule-1
IFN-γ	interferon-γ
IL-1	interleukin-1
JAK	Janus kinase
JNK	c-Jun N-terminal kinase
LPS	lipopolysaccharide
MAPK	mitogen-activated protein kinase
MCP-1	monocyte chemoattractant protein-1
MCs	macrophages
MDSCs	myeloid-derived suppressor cells
MMPs	matrix metalloproteinases
MR-proADM	mid-regional pro-ADM
MVD	microvessel density
N-cadherin	neural cadherin
NLRP3	NOD−, LRR− and pyrin domain-containing protein 3
NO	nitric oxide
OH	obese hypertensive
PDGF	platelet-derived growth factor
PI3K	phosphatidylinositol 3-kinase
PKA	protein kinase A
PLC-PKC	phospholipase C-protein kinase C
PVN	paraventricular nucleus
RAMP	receptor activity-modifying protein
RANKL	receptor activator of nuclear factor-kappa B ligand
RCC	renal cell carcinoma
SHP-2	Src homology phosphotyrosyl phosphatase 2
STAT	signal transducer and activator of transcription
TAMs	tumor-associated macrophages
TGF-β1	transforming growth factor-β1
Th1	T-helper 1
TME	tumor microenvironment
TMZ	temozolomide
TNF	tumor necrosis factor
Treg	regulatory T cells
TRPV2	transient receptor potential vanilloid 2
TSCC	tongue squamous cell carcinoma
UCP1	uncoupling protein 1
VCAM-1	vascular cell adhesion molecule-1
VE-cadherin	vascular endothelial cadherin
VEGF	vascular endothelial growth factor
